# The Suppressive Effect of Mamiran Cream on Atopic Dermatitis-Like Skin Lesions In Vivo

**DOI:** 10.1155/2021/2854238

**Published:** 2021-11-30

**Authors:** Kailibinuer Aierken, Yuqing Luo, Maitinuer Maiwulanjiang, Tao Wu, H. A. Aisa

**Affiliations:** ^1^State Key Laboratory Basis of Xinjiang Indigenous Medicinal Plants Resource Utilization, Xinjiang Technical Institute of Physics and Chemistry, Chinese Academy of Sciences, Urumqi 830011, China; ^2^University of the Chinese Academy of Sciences, Beijing 100039, China

## Abstract

**Background:**

The Chinese herbal formula Mamiran cream (MMC) has been known for its ameliorative effects on diverse skin diseases, such as eczema. Atopic dermatitis (AD; eczema) is a chronic recurrent skin disease dominated by T-helper type 2-driven inflammation (Th2).

**Objective:**

In this study, the inhibitory effect of MMC on AD was investigated in vivo.

**Methods:**

An animal model was established by sensitization with 2,4-dinitrochlorobenzene (DNCB) on the skin of SD rats. Cutaneous administration of MMC was applied, and its mechanism of action was investigated via RT-PCR and IHC assay.

**Result:**

Our data showed that topical application of MMC reduced the skin severity scores and alleviated the histological changes. Furthermore, immunohistochemical analysis demonstrated that MMC significantly decreased the levels of Th2 cytokine IL-5 and IL-4Ra in the skin lesion. In addition, it was demonstrated that MMC downregulated the mRNA expression of TNF-*α*, IL-1*β*, IL-6, IL-10, and TLR4. Moreover, MMC inhibited the activation of NF-*κ*B, JNK1, and STAT6 pathways in skin lesions.

**Conclusions:**

Our findings suggest that MMC exhibits the inhibitory effect on AD, suggesting that MMC may be a potential therapeutic agent for this atopic disorder.

## 1. Introduction

Eczema (AD) is a chronic inflammatory skin disorder characterized by pruritic eczematous lesions. It reduces the quality of life and increases healthcare. In recent decades, according to a recent survey in China, the prevalence of AD in children was approximately 0.70%, while it was approximately 8.3% in adolescents [1]. The pathogenesis of AD has been attributed to the complex interaction of pharmacological disorders, genetic and environmental factors, altered skin barrier defects, and the immunological system [2]. The main cause of AD is not fully delineated, and we and others have shown that the onset of AD seems to be more accurately caused by an imbalance between type-1 and type-2 helper T cells (Th1/Th2) in the immune system, and the factors induce Th2 cells to produce Th2 cytokines such as interleukin (IL)-4, IL-5, IL-10, and IL-13 and high IgE levels. Patients with AD commonly have elevated Th2-mediated inflammatory cytokines, such as interleukin (IL)-4, IL-5, and IL-13 during the acute and chronic stages. Th2 cytokines have direct effects on skin cells, such as keratinocytes. The affected keratinocytes produce proinflammatory cytokines and chemokines that induce the invasion of immune cells into inflammatory skin lesions. For instance, the expressions of inflammatory mediators such as nuclear factor-*κ*B (NF-*κ*B) are related to the severity of inflammation of AD [3]. Besides, in the immune milieu of AD, the enhancement of Th2-cell proliferation and their release of various cytokines through the JAK-STAT pathway may be the key factors of AD inflammatory responses. Long-term controllers, such as steroids, antihistamines, or calcineurin inhibitors, have been commonly used as effective therapeutic agents [4]. Steroids reduce all Th cell functions and are extensively used as immunosuppressants [5]. However, the prolonged use of steroid has significant adverse effects, which include thinning of the skin, leading to cracking, and bleeding [6–11]. Traditional Chinese medicine (TCM) has been proven to be potential source of therapeutic agents for preventing and treating inflammatory skin diseases in clinical studies. Findings from a systematic study on skin diseases show that Chinese herbal medicines alleviate AD and do not cause serious adverse effects [1, 12–14].

Several herbal prescriptions of TCM have been applied to treat inflammatory diseases for hundreds of years [15]. Mamiran cream (MMC) is a famous prescription of TCM that has been used in Chinese clinical practice for the treatment of various skin diseases, such as eczema. The formula composed of the stem of *Coptis chinensis* Franch (*C. chinensis*), galls of *Quercus infectoria* Oliv (*Q. infectoria*), roots of *Rumex dentatus* Linn (*R. dentatus*), and petals of *Rosa rugosa* Thunb (*R. rugosa*), at the ratio of 3 : 1.5 : 1.5 : 1. Among these ingredients, *C. chinensis* [16–18], *Q. infectoria* [18], *R. rugosa* [19, 20], and *R. dentatus* [21] have been proved to have anti-inflammatory and antiviral activities and have been applied for the treatment of inflammatory ailments, skin disorders, and infectious diseases. The main active compounds of MMC such as berberine and gallic acid have been verified to possess numerous biological activities. Previous studies show that berberine has a potent anti-inflammatory effect in vivo and in vitro [22] and can inhibit IgE production in human cells [23]. Another study showed that berberine directly suppressed histamine release by mast cells and IgE production by B/plasma cells and also inhibit STAT6, thereby possibly changing the process of inflammation [24]. In addition, gallic acid has well-documented anticancer [25], anti-inflammatory [26], and antibacterial effects [27]. A recent study has also reported that gallic acid could suppress the in vitro activation of AD-related basophils in allergic inflammation [28]. However, there is no research on the effects of MMC on AD in vivo and in vitro. Some of these research studies provide limited evidence for their inclusion in a formula for eczema. However, the scientific rationale for this formula is not confirmed [29].

Despite its long history of use, the anti-inflammatory and antiatopic mechanisms behind the effects of MMC have not been clarified. In our study, we evaluated the effects of MMC on DNCB-induced AD animal models. The anti-inflammatory and antiatopic properties of MMC were determined in a DNCB-induced AD model that aims to complement the shortcomings of using steroids in AD treatment. Besides, we studied inhibitory effects by MMC extract on the production of NO in LPS-induced RAW 264.7 cells.

## 2. Materials and Methods

### 2.1. Plant Materials and Chemicals

The HPLC grade acetonitrile and methanol were purchased from Merck KGaA (Darmstadt, Germany). The HPLC grade H_2_O was purchased from Wahaha Co., Ltd. (Hangzhou, China). Analytical grade phosphoric acid was obtained from Hong Sheng Chemical Reagent Co. (Tianjin, China). Reference compounds for berberine (bath number: 110713–201613) and gallic acid (bath number: 110831–201605) were obtained from the Chinese Food and Drug Accreditation Institute. RAW 267.4 cells were purchased from the Center of Cellular Resource, Chinese Academy of Sciences (Shanghai, China), and lipopolysaccharide (LPS), 3-(4,5-dimethylimidazol-2-yl)-2–5 diphenyltetrazolium bromide (MTT) and dimethylsulfoxide (DMSO) were purchased from Sigma Chemical Co. (St. Louis, Mo, USA). Dulbecco's modified Eagle's medium (DMEM) and fetal bovine serum (FBS) were produced by Gibco BRL (Grand Island, NY, USA). Liquid paraffin (20190702), glycerin (20190307), glyceryl monostearate (105620190501), stearic acid (20190612), triethanolamine (20190401), white Vaseline (20190301), ethyl hydroxybenzoate, sodium dodecyl sulfate (101420180508), and propylene glycol (20190501) were obtained from Hong Sheng Chemical Reagent Co. (Tianjin, China).

### 2.2. Preparation of MMC and Determination of Chemical Property of MMC Extract by HPLC

#### 2.2.1. Preparation of MMC Extract

All the herbal constituents of MMC were purchased from Xinlvbao Medicine (Xinjiang, China). *C. chinensis* (batch number: 20190500), *Q. infectoria* (batch number: MSZ-YP-171030), *R. rugosa* (batch number: 20150402), and *R. dentatus* (batch number: 180501) were identified by Dr. Chun fang Lu, and a voucher specimen (*Q. infectoria*, no. WY02260, *R. dentatus* WY-02661, *C. chinensis*, no. WY02662 and *R. rugosa* WY-02663) has been stored in the Xinjiang Technical Institute of Physics and Chemistry, Urumqi, Chinese Academy of Sciences. The medicinal composition and weight ratios of these herbs in the formula are shown in Table 1. The water and ethanol extracts of MMC were prepared as follows. *R. rugosa* and *Q. infectoria* were mixed together in proportion and were extracted with water for 1.5 h. *C. chinensis* and *R. dentatus* were extracted with 70% ethanol for 1.5 h. The extraction process was repeated three times, and the supernatant was collected.

#### 2.2.2. Conditions of HPLC

The MMC was extracted and filtered to make test liquid for injecting into the Agilent HPLC1260 liquid chromatography system with a diode array detector. Chromatographic separation of these analytes was performed on a Thermo C18 column (4.6 × 250 mm, 5 *µ*m). The mobile phase consisted of solvent A (acetonitrile) and solvent B (potassium phosphate monobasic, pH = 4) [30]. The gradient program was performed from 0 to 30% A in 0–20 min, 30–60% A in 20–23 min, 60–60% A in 23–28 min, and 60–30% A in 28–31 min, with a UV detector wavelength of 254 nm for standardizing berberine at room temperature, and the mobile phase consisted of methanol (as solvent C) and 0.3% phosphoric acid (as solvent D). The gradient elution was performed from 0 to 5% C in 0–20 min, 5–80% C in 20–23 min, 80–80% C in 23–28 min, and 80–5% C in 28–31 min, with a detection wavelength of 354 nm for standardizing gallic acid at room temperature.

#### 2.2.3. RAW 264.7 Cell Culture

RAW 264.7 cells were placed in DMEM supplemented with 10% fetal bovine serum, 100 U/mL penicillin, and 100 mg/ml of streptomycin at 37°C in a humidified CO_2_, there were added different concentrations of MMC extract, and they were incubated for 18 h with 1 mg/ml LPS.

#### 2.2.4. MTT Assay

Cells were plated in 96-well dishes, and after 24 h, various concentrations of MMC extract were added and incubated for 24 h. The cell viability was determined by an MTT method according to the method described by Xu et al. [14] .

#### 2.2.5. NO Production Assay

The determination of NO in cell cultures was performed according to Xu et al. [14]. In brief, cultured RAW 264.7 cells (1 × 10^5^ cells/mL) in 96-well dishes were pretreated with different concentrations of MMC for 1 h and stimulated with LPS (l *μ*g/mL) at 37°C for 18 h in medium.

### 2.3. Atopic Dermatitis Model and MMC Treatment

#### 2.3.1. Experimental Animals

6- to 7-week-old specific pathogen-free (SPF) SD rats (weighing 160–200 g) were obtained from the Xinjiang Laboratory Animal Center (Xinjiang Ethics Committee on Animal Experimentation, China, production certificate no: SCXK2018-0002). The animals were housed in a controlled room (temperature of 23 ± 2°C, humidity of 50 ± 10%, and 12 h/12 h dark and light cycle), and standard water and food were available. Animal experiments were approved by the Xinjiang Institute of Traditional Chinese Medicine Ethics Committee, and all procedures involving animals were conducted in accordance with the guidelines on ethical use.

#### 2.3.2. Formulation of MMC

The preparation method of the vehicle is as follows: the oil phase: liquid paraffin 100 g, glycerol monostearate 40 g, white Vaseline 70 g, and stearic acid 120 g were mixed and heated to 80°C. The aqueous phase: ethyl hydroxybenzoate 1.5 g, sodium dodecyl sulfate 4 g, propylene glycol 120 g, triethanolamine 4.5 g, and sodium ascorbate 4 g were mixed and heated to 80°C. Then, the oil phase was gradually added to the aqueous phase and stirred until it condensed to form the cream base. The 1,000 g MMC cream was prepared, and dexamethasone (DEX) was used for the positive control.

#### 2.3.3. Atopic Dermatitis Model Treatment

For in vivo experiments, DNCB was used as a sensitizer for establishing the animal model of AD in SD rats. Animals were sensitized with 100 *μ*L of 7% DNCB in acetone for three days. After 7 days from the first induction, 200 *μ*L of 0.7% DNCB was painted onto the dorsal skin of the rats for challenge, once a week for 6 weeks. The 6- to 7-week-old SD rats were randomly divided into 4 experimental groups (control group, DNCB-sensitized group, MMC-treated group, and DEX-treated group) of 5 rats per group. MMC (0.13, 0.25, and 0.5 g/rat, one time every day) and DEX (0.30 g/rat, one time every day) were dorsally administered for 2 weeks (days 7–21). Later, atopic dermatitis was challenged. As a control, equal amounts of normal saline were dorsally administered in the same manner. The total clinical severity of AD was calculated as the sum of the individual skin symptom scores assessed as 0 (none), 1 (mild), 2 (median), and 3 (severe) for each SD rat of the experiment according to the method described by Lee et al. [31]. In addition, the body weight of each animal was weighed weekly, and the difference among groups was compared. Immediately after 2 weeks of drug treatment, animals were made to fast for 12 h and euthanized with pentobarbital. Organs (spleen and thymus) were collected and weighed.

#### 2.3.4. Histological Analysis

At the end of the experiments, skin tissue samples were taken from the dorsal skin of each rat, fixed in 10% formalin, embedded in paraffin wax, and serially sectioned at 0.2 to 0.5 *μ*m in thickness. The section was stained with hematoxylin and encoded and determined under a light microscope by a pathologist.

#### 2.3.5. Immunohistochemistry (IHC)

According to the method described by Tang et al. [32], first, paraffin-embedded skin tissue samples were dewaxed with xylene, dehydrated in a gradually diminishing concentration of ethanol, and then rehydrated with water. Then, antigen retrieval was performed in phosphate-buffered saline (PBS) 3 times for 5 min each time in the microwave. Endogenous peroxidase activity was subsequently blocked by incubation in 3% H_2_O_2_ for 30 min and then incubated in 5% BSA in PBS blocking buffer to block nonspecific antigen at room temperature. Subsequently, slices were incubated with the appropriate primary antibodies (1 : 200) for 24 h in a 4°C humidified chamber. After washing with PBS, the slices were linked with a horseradish peroxidase-labeled goat anti-rabbit polyclonal antibody for 30 min at 37°C. Finally, the sections were then nuclear counterstained with hematoxylin.

#### 2.3.6. Real-Time Quantitative Polymerase Chain Reaction

Total RNA was extracted from dorsal skin of SD rats using TRIzol^®^ Reagent (Invitrogen, CA, USA). The total RNA template was reverse-transcribed into cDNA synthesis using SuperScript II reverse transcriptase (Invitrogen). The cDNA aliquots were amplified on SYBR Premix Ex TaqTM Kit (Takara Biotech) using GoTaq DNA polymerase (Promega), and the gene primers are shown in Table 2. The transcript levels were quantified by using the 2 − ΔΔC_t_ value method with beta tubulin as internal normalization.

### 2.4. Statistical Analysis

Data values of the results were shown in mean ± SD of three determinations. Statistical analysis was performed using Prism 7.0 (GraphPad software, Inc., San Diego, CA). Comparisons were made using analysis of variance (ANOVA), and *p* value <0.05 was considered as significant. All experiments were done in triplicate, and the data are plotted as the mean ± SD of triplicate determinations.

## 3. Results

### 3.1. Yield of Crude Extracts and Determination of Marker Compounds of MMC by HPLC

**Figure 1:**
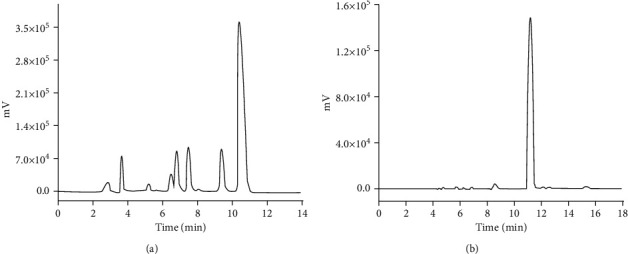
HPLC chromatograms of berberine and gallic acid in Mamiran cream (MMC). (a) HPLC chromatogram of berberine in MMC. (b) HPLC chromatogram of gallic acid.

To obtain the best extraction yield and high amount of active ingredients from herbal materials, the extraction technology was optimized including extraction solvent, extraction time, mass liquid ratio, and extraction method. The two major components in MMC were analyzed by HPLC (Figure 1). The components of the MMC were gallic acid (6.56%) and berberine (2.93%), respectively.

### 3.2. Effect of MMC on RAW 264.7 Cell Viability

**Figure 2:**
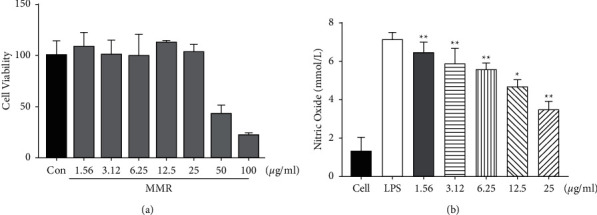
Cytotoxicity of MMC in RAW 264.7 cells. Cells were treated with different concentrations of MMC for 24 h, and viability was assayed by the MTT assay. (a) MMC at 25 *μ*g/mL was not cytotoxic. Effects of MMC on LPS-induced NO production in LPS-induced RAW 264.7 cells. Cells were incubated in the presence of MMC or in combination with 1 *μ*g/mL LPS for 18 h. The culture supernatant was analyzed for NO (b) production. Values are mean ± SD from three independent experiments. ^*∗*^*p* < 0.05 and ^*∗∗*^*p* < 0.01 versus LPS-induced group (one-way ANOVA).

According to the MTT experiment, MMC did not show cytotoxicity under 25 *μ*M concentration treatment in RAW 264.7 cells (data shown in Figure 2(a)).

### 3.3. Effect of MRR on NO Production

In this study, we investigated the change in NO production by MMC in RAW 264.7 cells. NO production increased in the LPS-treated group. However, it was reduced when MMC was pretreated before LPS treatment in RAW 264.7 cells (data shown in Figure 2(a).

### 3.4. MMC Reduces the Severity of Atopic-Like Lesions and Decreases Histopathological Features in DNCB-Induced AD Rats

**Figure 3:**
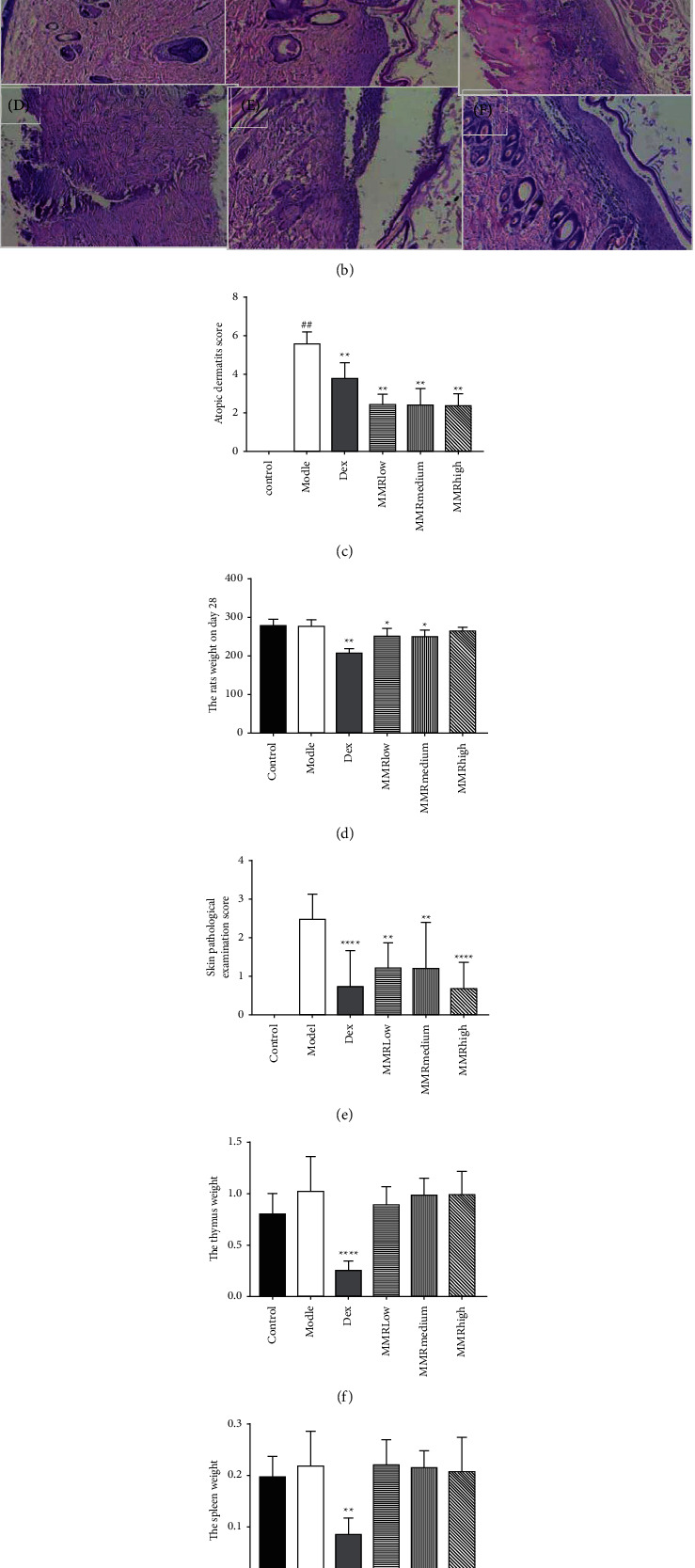
MMC attenuated DNCB-induced clinical symptoms. The photographs were obtained before the rats were sacrificed (a). Rat dorsal skins were removed after the experiment. The dorsal skin lesions were stained with hematoxylin and eosin (b). In (a, b),control group (A), DNCB-induced group (B), DEX-treated group (C), MMC low-dose group (D), MMC medium-dose group (E), and MMC high-dose group (F), The clinical severity of inflammation of each rat dorsal skin lesion was evaluated (c). Rats mean body weight (d). Skin pathological examination score (e). The thymus weight and the spleen weight (f, g) were measured by using an electric scale. Data are presented as mean ± SD from five fields in each group (*n* = 5). ^#^*p* < 0.05;  ^##^*p* < 0.01, compared to control. ^*∗*^*p* < 0.01,  ^*∗∗*^*p* < 0.001, compared to DNCB-induced group.

To investigate the effect of MMC on AD in our animal model, we sensitized the SD rats' dorsal skin with DNCB allergen to induce clinical characteristics of AD, such as dryness, erythema, erosion, and edema.  Figure 3(a) indicates that treatment of MMC ameliorated these phenotypes in skin lesions. Dermatitis scores and skin pathological examination scores in each group were calculated. MMC-treated group and DEX-treated group had decreased clinical skin severity scores compared to the DNCB-induced group. Thus, MMC treatment reduced dermatitis scores (Figure 3(c)). The body weight and the index of the spleen and thymus of the MMC-treated group did not show significant change. Compared with the model group, the weight of the spleen and thymus in the DEX group was significantly decreased, and the difference was statistically significant (*p* < 0.01). The positive control, DEX acetate, belongs to glucocorticoids and has an immunosuppressive effect. Large area topical administration can significantly reduce the weight and index of spleen and thymus in SD rats, which is related to the pharmacological action of adrenal corticoids (Figures 3(d), 3(f), and 3(g). Hematoxylin and eosin experiment shows that the skin lesion tissue of control rats reflected AD model and a marked thickening of the cuticle, epidermis, and dermis, accompanied by increased infiltration of inflammatory cells into the skin tissue (Figures 3(b) and 3(e)). However, medium and high dosage groups of MMC inhibited the pathologic changes including hyperkeratosis and parakeratosis, resulting in a histological presentation very similar to that of the control group.

### 3.5. Expression of IL-4R*α*, JAK1, p-STAT6, and IL-5 Evaluated by Immunohistochemistry

**Figure 4:**
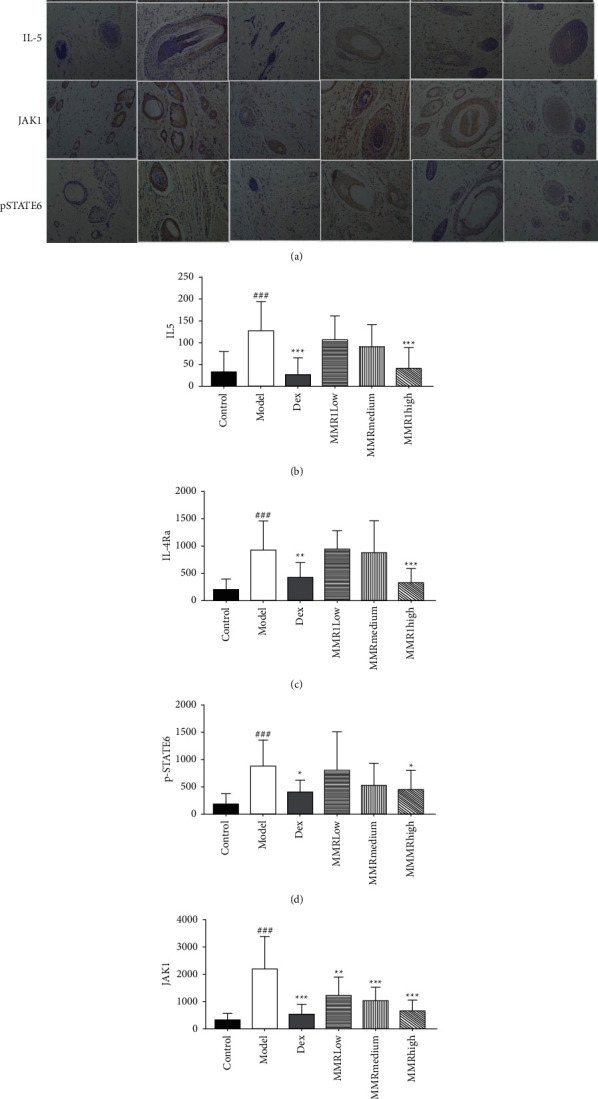
IL-4R*α*, JAK1, p-STAT6, and IL-5 expression levels in dorsal skin tissue were detected by immunohistochemical staining (a). The figures are representative of each group of mice (*n* = 5). The images were obtained at ×200 magnification. Quantification of IL-4R*α*, JAK1, p-STAT6, and IL-5 protein levels (b–e). Data are presented as mean ± SD from five fields in each group (*n* = 5). ^#^*p* < 0.05;  ^##^*p* < 0.01, compared to control; ^*∗*^*p* < 0.01,  ^*∗∗*^*p* < 0.001, compared to DNCB-induced group.

Immunostaining for IL-4R*α*, JAK1, p-STAT6, and IL-5 was further performed in dorsal skin tissues to localize the proteins in specific cell types. IL-4R*α*, JAK1, p-STAT6, and IL-5 could be observed in the nucleus and cytoplasm.  Figure 4(e) indicates that the expressions of IL-4R*α*, JAK1, p-STAT6, and IL-5 were all significantly elevated in the DNCB-induced group compared to the control group (*p* < 0.001). The MMC-treated group and DEX-treated group had significantly decreased levels of IL-4R*α*, JAK1, p-STAT6, and IL-5 expression as compared with the DNCB-induced group (*p* < 0.05 or *p* < 0.01 or *p* < 0.001) while MMC high-dose group showed a higher suppressive effect on IL-4R*α* cell infiltrations than the DEX-treated group (*p* < 0.05 or *p* < 0.01) (Figure E). Those results assumed that MMC might inhibit atopic skin inflammation by inhibition of Th2 expression.

### 3.6. MMC-Suppressed DNCB Increased mRNA Level of Inflammatory Cytokine in SD Rats

**Figure 5:**
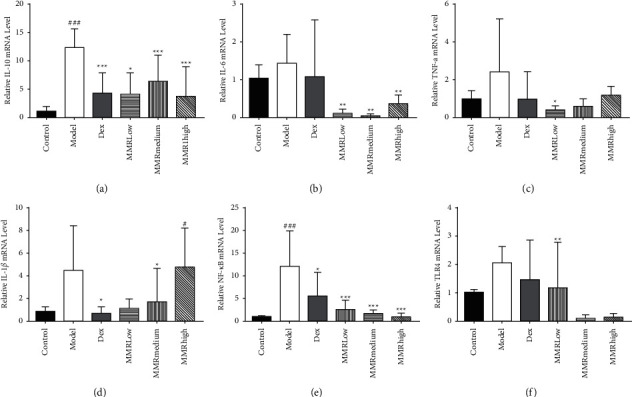
MMC decreased proinflammatory cytokines in the DNCB-induced rat skin tissue. The mRNA expression levels of IL-10 (a), IL-6 (b), TN-*α* (c), IL-1*β* (d), NF-*κ*Bp65 (e), and TLR4 (f) were determined using real-time PCR. Values are means ± SD (*n* = 5). ^*∗∗*^*p* < 0.01,  ^*∗∗∗*^*p* < 0.001.

The mRNA levels of inflammatory mediators such as TNF-*α*, IL-1*β*, IL-6, and anti-inflammatory cytokine IL-10 were also increased in the DNCB-sensitized group (*p* < 0.05) (Figures 5(a)–5(f)) and, however, decreased in the MMC-treated group (*p* > 0.05). Therefore, the mRNA level of TLR4 was significantly higher in the DNCB-sensitized group. The MMC-treated group showed a nonsignificant decrease in TLR4 mRNA levels, and it was found that MMC affected the NF-*κ*Bp65 pathway. These results suggest that MMCs inhibit the expression of TNF-*α*, IL-1*β*, IL-6, TLR4, and NF-*κ*Bp65 in dorsal skin.

## 4. Discussion

Mamiran cream (MMC) is a famous Chinese herbal prescription, which is traditionally used to treat skin diseases for years in Xinjiang, China, and it shows a potential therapeutic effect on eczema. In our study, we verified that topical treatment of MMC decreased the infiltration of inflammatory cells and epidermis hypertrophy in the skin of DNCB-induced rats. In addition, the treatment of MMC reduced the thymus and spleen weights that were increased by DNCB sensitization. Furthermore, immunohistochemical analysis demonstrated that MMC significantly reduced the levels of Th2 cytokine, including IL-5 and IL-4Ra in the skin lesion. Also, MMC downregulated the mRNA expression of proinflammatory cytokines TNF-*α*, IL-1*β*, IL-6, IL-10, and TLR4 and suppressed the NF-*κ*B pathway in skin lesions. Moreover, MMC extract inhibited the production of nitric oxide (NO) in LPS-induced RAW 264.7 cells in vitro.

AD is one of the most and common relapsing inflammatory skin diseases, which is characterized by Th1/Th2 imbalance [3]. Dorsal application of DNCB on the backs of rats induced many of the cutaneous histopathological signs of eczematous skin lesions within a month. AD-like skin lesions in animal models were similar to those in AD patients. We observed a high degree of infiltration of inflammatory cells and epidermis hypertrophy in the skin tissue lesions of DNCB-induced SD rats. After 4 weeks of treatment with MMC, the symptoms of AD-like skin lesions were significantly improved.

According to previous data from human and murine studies, AD is a Th2-dominant inflammatory disease in acute atopic eczema followed by Th1 involvement at the chronic stage [33]. Th1 and Th2 responses are balanced by cytokine regulation, and the imbalance of Th1/Th2 cells plays a pivotal role in the immune pathway of AD [34]. Jin et al. [35] proved that Th2 cytokine IL-4 and IL-5 levels in the acute phase of dorsal AD-like skin lesions were significantly elevated than in the normal skin tissue. The available studies suggest that the development of AD is partly due to the imbalance of immune response mediated by activation of JAK-STAT signaling. The JAK-STAT pathway is activated by IL-4 and IL-5, which play a vital role in AD-related skin lesions by alleviating proinflammatory cytokines and angiogenic mediators. The previous experimental study demonstrated that [34, 36] IL-4 induces heterodimerization of its receptor (IL-4R) accelerating tyrosine phosphorylation of IL-4R*α* chain and activation of JAK1 [37]. Besides, Th2-associated cytokine IL-5 activates eosinophils attracted to the skin, further exacerbating the clinical lesions of AD. Based on the above findings, we performed immunohistochemical analyses in the skin and the data demonstrated that MMC suppressed expression of IL-4R*α* and IL-5 in the skin tissue of DNCB-sensitized rats (Figure 4). In addition, MMC significantly inhibited the phosphorylation of JAK1 and STAT6 expressions. Therefore, we hypothesized that MMC treatment benefits the alleviation of typical clinical signs of AD-like skin lesions, which is by reducing cytokine expression levels and inhibiting downstream pathways of cytokines.

Inflammation cytokines, namely, TNF-*α*, IL-6, IL-10, and IL-1*β*, are involved in AD development and progression. Our study confirmed that TNF-*α*, IL-6, IL-10, and IL-1*β* were markedly elevated in DNCB-induced skin as compared to those in the normal skin tissue. We also observed a strong inhibitory effect of MMC on these inflammatory cytokines. These effects for attenuating immune responses by regulating Th1/Th2 balance are partially related to the reduction in inflammatory properties. Furthermore, to investigate the mechanism underlying the inhibitory effects of MMC on inflammation, we assumed the inflammatory effects of MMC on the NF-*κ*B pathway. The obtained results show the production of inflammatory cytokines, such as IL-1*β*, IL-6, and TN-*α*, is NF-*κ*B dependent in vivo and in vitro [38]. The suppression of TLR4 triggers NF-*κ*B signaling pathway and reduces the inflammatory response, which may regulate the expression of TNF-*α* to alleviate the clinical signs in the lesional skins tissue [39, 40]. In this study, NF-*κ*B activity and the expression of TLR4 were significantly increased in DNCB-induced skin tissue. However, dorsal administration with MMC markedly suppressed NF-*κ*B activation. MMC exerts partially mediate the Th1/Th2 immune response by regulation and inhibition of the NF-*κ*B/TLR4 signaling pathway.

Based on the above analysis, we hypothesized that topical application of MMC can ameliorate the pathological features and inflammation of AD lesions, including skin lesion severity, histopathologic indexes, dermatitis score, Th2-related inflammatory cytokines, and immune response of DNCB-induced rats. Meanwhile, the serum level of IL-4, the blood cell population, and weight of liver were also calculated at the end of the experiment. Compared with the DNCB-induced rats, MMC treatment groups had no significant changes (data were not shown). So, it can be hypothesized that the dorsal administration of MMC had no systemic effects on AD.

In conclusion, the result obtained in our research assessed that MMC administration can reduce inflammatory cell infiltration, decrease the expression of inflammatory cytokine, downregulate IL-4R*α* and TLR4 expressions, and inhibit NF-*κ*B, JAK1, and pSTATE6 activation. Therefore, MMC may be the therapeutic option without side effects for the treatment of eczema or other inflammatory skin diseases.

## 5. Conclusions

MMC dorsal administration alleviated the clinical features of AD. Furthermore, MMC decreased the levels of Th2 cytokine IL-5 and MMC treatment has a remarkable downregulatory effect on IL-4Ra, TNF-*α*, IL-1*β*, IL-6, IL-10, and TLR4 expressions in skin lesions. Our findings also indicated that MMC can ameliorate the immune response by inhibiting activation of the NF-*κ*B, JNK1, and STAT6 pathways in AD lesions. These observations suggest that MMC may, therefore, be a potential drug for the treatment of eczema.

## Figures and Tables

**Table 1:** The composition of Mamiran cream (MMC)

Latin name	Chinese herbal name	Medicinal parts	Grams	Weight ratio
*Coptis chinensis* Franch	Huanglian	Stem	120	42.85
*Quercus infectoria* Oliv	Moshizi	Galls	60	21.42
*Rumex dentatus* Linn	Tudahuang	Roots	60	21.42
*Rosa rugosa* Thunb	Meiguihua	Petals	40	14.28
Total amount			280	100

**Table 2:** Primers utilized for real-time PCR

Gene	Forward	Reverse
*β*-Actin	CCCATCTATGAGGGTTACGC	TTTAATGTCACGCACGATTTC
TLR4	TCCTTTCCTGCCTGAGACCA	TGTCTCAATTTCACACCTGGAT
NF-*κ*b	TGTATTTCACGGGACCTGGC	CAGGCTAGGGTCAGCGTATG
IL-1*β*	AAATGCCTCGTGCTGTCTGA	TTGGGATCCACACTCTCCAG
TNF-*α*	GTCCCAACAAGGAGGAGAAGTT	CTCCGCTTGGTGGTTTGCTA
IL-6	GCAAGAGACTTCCAGCCAGT	TGCCATTGCACAACTCTTTTC
IL-10	CTGCTGGATGACTTTAAGGGTT	GCAGGGCAGAAAACGATGAC

## Data Availability

All data generated or analyzed during this study are included in this published article.
